# The burden of hyaline membrane disease, mortality and its determinant factors among preterm neonates admitted at Debre Tabor General Hospital, North Central Ethiopia: A retrospective follow up study

**DOI:** 10.1371/journal.pone.0249365

**Published:** 2021-03-30

**Authors:** Binyam Minuye Birihane, Wubet Alebachew Bayih, Abebaw Yeshambel Alemu, Demeke Mesfin Belay, Asmamaw Demis

**Affiliations:** 1 College Health Sciences, Debretabor University, Debretabor, Ethiopia; 2 Department of Nursing, College Health Sciences, Woldia University, Woldia, Ethiopia; Kobe University Graduate School of Medicine School of Medicine, JAPAN

## Abstract

**Background:**

Hyaline membrane disease (HMD) is a leading cause of morbidity and mortality in preterm newborn babies. Though, there are studies related to Hyaline membrane disease inclusive of all neonates, studies related to the burden among preterm neonates were limited. In addition, increasing neonatal mortality in Ethiopia could be related to increase in the burden of hyaline membrane disease among preterm neonates. Therefore, this study was aimed to assess the burden of hyaline membrane disease, mortality and its associated factors among preterm neonate admitted at neonatal intensive care unit, North Central Ethiopia.

**Methodology:**

An institution-based retrospective follow-up study was conducted among 535 preterm neonates admitted at neonatal intensive care unit from January 1, 2014-December 30, 2017. Data were entered into EPi-data 4.2.0.0 and transferred to STATA version 14 statistical software for statistical analysis. Binary logistic regression was used for the analysis. All variables with P-value < 0.25 during bi-variable analysis were considered for multivariable logistic regression. Level of statistical significance was declared at P value ≤0.05.

**Results:**

In the current study, proportion of hyaline membrane disease was 40% (95% CI; 35.8, 44.3) of which 49.5% died. Preterm neonate born with Gestational age of less than 34 weeks of age (Adjusted odd ratio (AOR = 2.64; 95 CI: 1.49, 4.66)), 5^th^ minute Apgar score less than 7 (AOR = 2.2; 95% CI: 1.20, 4.07), and newborn with birth weight of less than 1500 gram (AOR = 2.4, 95% CI: 1.3, 4.3) were predictors of hyaline membrane disease.

**Conclusions:**

The mean gestational age (±) was 33.46 (±2.55) weeks. The incidence of hyaline membrane disease among preterm admissions was high. Preterm neonate born with gestational age of less than 34 weeks of age, asphyxiated newborns and newborn with birth weight of less than 1500 gram were predictors of hyaline membrane disease. So, emphasis should be given on early screening, follow up and timely interventions for preterm neonate.

## Background

More than a third of newborn deaths are related to preterm birth complications. Hyaline membrane disease (Neonatal respiratory distress syndrome (NRDS) is one of the commonest health problem encounter in preterm neonates and typically worsen within the first 48 to 72 hours [1, 2]. It is diagnosed based on the presence of one or more of the following signs: an abnormal respiratory rate, expiratory grunting, nasal flaring, chest wall recessions and thoracic-abdominal asynchrony with or without cyanosis [3–5].

Being born preterm increases risk of dying because of immature organs [6, 7]. Hyaline membrane disease (HMD) is the leading cause of mortality and morbidity in preterm newborn babies [8–12], 45% of cause of death in Ethiopia [13], 46.9% in Nigeria [14], 12.8% in Poland [15]. In addition, the burden of NRDS among neonates reported as 52.9% of total admission in Egypt [16], 26.2% in Nigeria [14], 34.7% in Iraq [17], 57% in north India [18], 23.5% in nepal [19],11.9% in Jimma [20].

About three-quarter of preterm neonates could survive, if they had access to early screening and care [21]. Even though, provision of advanced care for preterm neonates, and mothers; the world health recommendations of surfactant replacement therapy for preterm neonates and corticosteroid administration for mother at risk of preterm birth [22–27], care during pregnancy and childbirth [28, 29], and high flow of nasal oxygen therapy [30]; the burden of HMD related morbidity and mortality is increasing. Preterm mortality is continuing to be one of the global agendas in the sustainable development goals [31].

Based on different studies; gestational age [32], vitamin D status [33], male babies, previous birth of a baby with HMD, cesarean delivery [3, 34, 35], perinatal asphyxia, cold stress, perinatal infection, infants of diabetic mothers [36, 37],abnormal placental implantation [38, 39] were reported as risk factors for hyaline membrane disease.

Though, there are studies related to HMD inclusive of all neonates, studies related to the burden among preterm neonates were limited. In addition, increasing neonatal mortality in Ethiopia could be related to increase in the burden of hyaline membrane disease. Therefore, thestudy was aimed atassessing the burden of hyaline membrane disease, mortality and its associated factors among preterm neonate admitted at neonatal intensive care unit, North Central Ethiopia.

## Methodology

### Study design, period, setting, and population

An institutional based retrospective follow-up study was conducted among 535 preterm neonate with a study period from January 1, 2014-December 30,201. All preterm neonates admitted at Debre Tabor General Hospital during the study period were included. Debre Tabor town is found 666 km far from Addis Ababa, capital city of Ethiopia. The hospital receives a patient with severe disease referred from the nearby Primary Hospitals and Health Centers which is also true for neonatal care. The hospital provide health care service for more than 2.4 million populations in its catchment area. The hospital has a Neonatal Intensive Care Unit (NICU) with a total of 21 neonatal beds and approximately 1,159 neonates were admitted per year. Most of the neonatal admissions were associated with prematurity [40].

### Sample size determination and sampling procedure

A total of 614 preterm neonates admitted during the study period. Of these, medical charts were not available for 25 neonates and 54 charts were excluded due to data incompleteness. Finally, 535 preterm neonates were involved in the study. A list of medical record number was found from Neonatal registration book. Then, charts were found from medical record office by using medical chart number.

### Data collection

Data were collected based on the structured data abstraction sheet from medical charts. Abstraction sheet includes maternal socio-demographic, obstetric, and neonatal-related variables by considering hyaline membrane as an outcome variable.

### Variables

#### Dependent variable

Hyaline membrane disease (Yes, No).

#### Independent variables

✓ Socio-demographical characteristics of the mother (Age of the mother, Residence of the mother
✓ Obstetric related factors(Gestational age, Gravidity, Parity, ANC follow up, medical/surgical complications (Preeclampsia/Eclampsia, APH, Chorioamnionitis)
✓ Intrapartum factor (Preterm premature rupture of membrane, place of delivery, Weight of the infant at delivery, multiple pregnancies, mode of delivery)
✓ Neonatal related factor (Sex of the neonate, Apgar score, and birth order)

### Operational definitions

Maternal complication was considered if mother had an obstetric hemorrhage, puerperal sepsis and pyrexia, prolonged labor, eclampsia and preeclampsia, mal-presentation and mal-position, premature rupture of membrane (PROM), cord prolapse, obstructed labor, cephalopelvic disproportion, emergency cesarean section, and retained placenta.

Hyaline membrane disease was diagnosed based on the presence of one or more of the following signs: an abnormal respiratory rate, expiratory grunting, nasal flaring, chest wall recessions and thoraco-abdominal asynchrony with or without cyanosis [3].

### Data quality control

Pretest was done on 5% of the sample size. One day training was given for data collectors and supervisors on data collection tools and data collection procedures. Completeness of each data collection tool had been checked on a daily base. Double data entry was done by two data clerks and consistency of the entered data was cross-checked.

### Statistical analysis

Data were entered, coded, cleaned, and checked by Epi Data version 4.2.0.0 and analyzed using STATA Version 14 statistical software. Descriptive statistics of included variables were presented by using tables and texts. Binary logistic regression was used to saw the association between each independent variable with a dependent variable. All variables with a p-value≤0.25 in the bi-variable analysis were entered into multivariable analysis. Variables with p values ± 0.05 in multivariable logistic regression model analysis was considered as statistically significant.

### Ethical considerations

Ethical clearance was obtained from the Institutional Health Research Ethics Review Committee of Haramaya University, Ethiopia. Then, official letter was written to Debre Tabor General Hospital for permission. The neonates’ name and medical record identification information were not collected. Since the data obtained from medical charts, all data were fully anonymized. All data extracted from the chart were kept strictly confidential.

## Result

### Sociodemographic and obstetric characteristics of the mother and neonate

The mean age with standard deviation (±SD) of mother was 27.56 (±6.54) years. Sixty-six percent, of mothers were in the age group of 20–34 years. The majority, (72.7%) of them were from the rural area. Seventy-seven percent, (77.1%) of mothers had ante-natal care (ANC) follow-up. Sixty-nine percent of preterm neonates were single births. More than half, 51.6% were male newborn with mean gestational age (±) of 33.46(±2.55) weeks (Table 1).

**Table 1:** Maternal and neonatal related characteristics who had preterm neonatal admission at Debre Tabor General Hospital NICU, Northwest Ethiopia, from January 1, 2014–December 30, 2017.

Variable	HMD		
	YES	NO	Total
	n (%)	n(%)	
Residence			
Urban	56 (26.1)	90 (28.0)	146 (27.3)
Rural	158 (73.8)	231(72.0)	389 (72.7)
Maternal age			
<20 yrs.	25(11.7)	61(19.0)	86(16.1)
20–34 yrs.	148(69.2)	205(63.9)	353(66.0)
>34yrs.	41(19.2)	55(17.1)	96(17.9)
ANC visit			
Yes	170(79.4)	242(75.4)	412(77.0)
No	44(20.6)	79(24.6)	123(23.0)
Number of ANC visit			
<4	157(98.1)	193(79.8)	350(84.9)
≥4	3 (1.9)	49(20.3)	52(15.1)
Parity			
Prim	73 (34.1)	138 (43.0)	211(39.4)
1–4 births	100 (46.7)	142(44.2)	242 (45.2)
≥ 5 births	41(19.2)	41(12.8)	82 (15.3)
Birth type			
Single	138 (64.5)	229 (71.3)	367(68.6)
Multiple	76(35.5)	92(28.7)	168(31.4)
Birth weight			
<1500gm	100 (46.7)	62(19.3)	162(30.9)
≥1500gm	113(52.8)	249(77.6)	362(69.1)
Gestational age			
<34wk	138(64.4)	101 (33.3)	239(44.2)
≥34wk	76 (35.5)	202(66.7)	278(52.0)
Birth order			
1^st^	63 (29.4)	128(39.8)	191(35.7)
≥2	151(70.6)	193(60.1)	344(64.3)
Preeclampsia			
Yes	40(18.7)	41(12.8)	81(15.1)
No	174(81.3)	280(87.2)	454(84.9)
Antenatal corticosteroid			
Yes	27(17.4)	31(8.9)	58(11.5)
No	128(82.9)	316(91.1)	444(88.5)
Sex of neonate			
Male	110(51.4)	166(51.7)	276(51.6)
Female	104(48.6)	155(48.3)	259(48.4)
Died			
Yes	106(49.5)	61(19.0)	167(31.2)
No	108(50.5)	260(81.0)	368(68.8)

_HMD-Hyaline membrane disease, ANC-Antenatal care, COR-Crude odd ratio, AOR-Adjusted Odd Ratio_

### Proportion of hyaline membrane disease

In the current study, the incidence of hyaline membrane disease was 40.0% (95% CI; 35.8, 44.3); among this 49.5% (106 per 214) were died.

### Predictors of hyaline membrane disease

Both bi-variable and multivariable logistic regression were undertaken. In binary logistic regression gestational age, number of antenatal care visit, parity, birth order, birth weight, maternal complications, APH, 5^th^ minute Apgar score and PPROM were significantly associated with HMD. Whereas, in multivariable analysis; Gestational age, 5th minute Apgar score and birth weight were statistically significant.

In this study, newborn with less than 34 weeks of gestation were 2.64 times more likely to develop hyaline membrane disease than neonates born greater than or equal to 34 weeks of gestation (AOR = 2.64; 95 CI:1.49,4.66). Similarly preterm neonate born with less than 7 APGAR score of 5^th^ minute were 2.2 times more likely to have hyaline membrane disease in contrast with newborns with APGAR score of greater than seven (AOR = 2.2; 95% CI:1.20,4.07).

Those preterm neonates born with birth weight of less than 1500 gram were 2.4 times more likely to have hyaline membrane disease than newborn with birth weight of greater than 1500 gram (AOR = 2.4,95% CI:1.3,4.3) (Table 2).

**Table 2:** Predictors of hyaline membrane disease admitted in NICU, Debretabor General Hospital, from January 1, 2014-December 30, 2017.

Variable	HMD			
	YES	NO	COR	AOR
	n (%)	n (%)		
Gestational age				
<34wk	138 (25.8)	101 (18.9)	3.96 (2.7, 5.7)	2.6 (1.5,4.7)*
≥34wk	76 (14.2)	202(41.1)	1	1
Number of ANC visit				
<4	157(38.1)	193 (46.8)	3.1(1.6,5.9)	1.31 (0.60, 2.87)
≥4	3 (3.2)	49 (11.9)	1	1
Parity				
Prim	73 (13.6)	138 (25.8)	1	1
1–4 births	100 (18.7)	142 (26.5)	1.33 (0.91,1.95)	0.53 (0.15, 1.79)
≥ 5 births	41 (7.7)	41 (7.7)	1.89 (1.13,3.17)	0.90 (0.24,3.46)
Birth order				
1^st^	63 (11.8)	128 (23.9)	1	1
≥2	151(28.2)	193 (36.1)	1.59 (1.10,2.30)	2.5 (0.72,8.86)
Maternal complication				
Yes	93 (17.4)	93 (17.4)	1.88 (1.31 2.71)	1.2 (0.65,2.18)
No	121(22.6)	228 (42.6)	1	1
5^th^ min APGAR score				
<7	59 (15.7)	33 (8.8)	3.14 (1.92,5.13)	2.2 (1.20,4.07)**
≥7	103 (27.4)	181 (48.1)	1	1
APH				
Yes	17 (3.2)	12 (2.2)	2.22 (1.04,4.75)	2.46 (0.69,8.81)
No	197 (57.8)	309 (36.8)	1	1
Birth weight				
<1500gm	100 (19.1)	62 (11.8)	3.55 (2.41,5.23)	2.4 (1.3,4.3)***
≥1500gm	113 (21.6)	249 (47.5)	1	1
PROM				
Yes	33 (6.2)	29 (5.4)	1.84 (1.08,3.13)	1.07 (0.46, 2.48)
No	181 (33.8)	292 (54.6)	1	1

_HMD-Hyaline membrane disease, ANC-Antenatal care, APH-Antepartum Hemorrhage, COR-Crude odd ratio, AOR-Adjusted Odd Ratio_

* Significant at P-value less than 0.005.

## Discussion

Hyaline membrane disease is one of the significant predictor of neonatal morbidity and mortality. In this study proportion of hyaline membrane disease was 40%, this is consistent with study conducted in Iraq, 42.2% [17], in Black lion Hospital, Ethiopia 42.9% [41]. But, higher than study conducted in Nigeria, 26.2% [14], in Bulgaria, 15% [42], in Nepal 17.8% [43], in Jimma Ethiopia 11.9% [20].This could be the current study include only preterm neonates. In addition, the study conducted in Bulgaria was conducted among preterm neonate whose mother took dexamethasone. However, proportion was lower than the study conducted, in Cameroon 47.5% [3], 54,3% in Poland [15], 54.7% Saudi Arabia [44], 49.6% in Egypt [45]. The possible reasons could be the study conducted in Poland was conducted among preterm births from PROM mother which may increase the burden. In addition, it could be a difference in the study setting, period, and socio economic difference between the countries.

In multi variable analysis those with gestational age of less than 34 weeks of age, asphyxiated newborns and newborn with birth weight of less than 1500gram were predictors of hyaline membrane disease. In the current study, newborn with less than 34 weeks of gestation were more likely to develop hyaline membrane disease than neonates born greater than or equal to 34 weeks of gestation. This is consistent with study conducted in China [46], and India [47].The reason could be as gestational age increase hormonal level of serum TSH levels increase which have an impact on lowering incidence of NRDS [48]. In addition, as the gestational age decrease the probability of being borne with low Apgar score increase [49], which increase HMD. Moreover, surfactant deficiency is more common in preterm neonate delivered before 34 weeks of gestation. Thus, identifying and providing critical care for preterm neonates with gestational age less than 34 weeks of gestation has to be practiced. In addition, non-natural surfactant administration should be considered for preterm neonates’ gestational age less than 34 weeks.

Preterm neonates born with 5^th^ minute Apgar score of less than 7 were more likely to have hyaline membrane disease in contrast with newborns with Apgar score of greater than seven. This is similar to studies conducted in UK [50], USA [51], Cameroon [3], China [46], and Adis Ababa [41]. This could be because of the fact that low Apgar score result in birth asphyxia and low level red cell mass which interfere with cardio-respiratory adaptation result in increases the risk of Hyaline membrane disease [52, 53]. Those preterm neonates born with birth weight of less than 1500 gram were 2.4 times more likely to have respiratory syndrome than newborn with birth weight of greater than 1500 gram. This is consistent with a study conducted in Italy [34].The rational could be low birth weight neonates have high risk to have immaturity of physiological and anatomical structures such as; deficient in surfactant, lack of subcutaneous tissue, large surface area to body mass ratio, and fragile capillaries in their brain [54]. Hence, deficiency in surfactant administration increases the risk of pulmonary edema which results in Hyaline membrane disease. To decrease the burden related to hyaline membrane disease the hospital should give emphasis on treatment strategies of HMD. Nowadays, the hospital is implementing treatment strategies for neonate with HMD such as nasal continuous positive airway pressure by mask, nasal prong, nasopharyngeal tube; high flow nasal cannula and oxygen supplement. However, the hospital is not implementing surfactant administration. Therefore, the hospital should give emphasis on early screening, update and integrating other treatment strategies (surfactant administration) for HMD neonates as per the protocol. Surfactant administration will be based a combination of clinical, radiological, or laboratory findings (neonates with clinical and radiographic evidence of RDS, neonates at risk of developing RDS (e.g. <32 weeks or low birth weight <1300g or neonates who are intubated, regardless of gestation, and requiring FiO_2_ >40%).

Since, the study is retrospective follow-up study important variables like surfactant administration, Vitamin D status of the newborn and placental abnormality were missed. Therefore, future researcher should do prospective studies by including those variables which may have key indicators for HMD.

## Conclusions

The incidence of hyaline membrane disease among preterm birth was high. Preterm neonate born with gestational age of less than 34 weeks of age, asphyxiated newborns and newborn with birth weight of less than 1500 gram were predictors of hyaline membrane disease. So, emphasis should be given on early screening, follow up and timely interventions of preterm neonates borne with low birth weight and low Apgar score.

## Supporting information

S1 File (DTA)

## List of abbreviations

AOR: Adjusted Odd Ratio
ANC: Antenatal Care
APH: Antepartum Hemorrhage
CI: Confidence Interval
DTH: Debre Tabor Hospital
HMD: Hyaline membrane disease
IRERC: Institutional Research Ethics Review Committee
LBW: Low Birth Weight
NICU: Neonatal Intensive Care Unit
PPROM: Pre labor premature rupture of membranes
NRDS: Neonatal Respiratory Distress Syndrome
SDG: Sustainable development goal
WHO: World Health Organization
